# Effectiveness of Stress Management Skill Training on the Depression, Anxiety and Stress Levels in Drug Addicts after Drug Withdrawal

**DOI:** 10.5812/ijhrba.10695

**Published:** 2013-09-20

**Authors:** Zahra Habibi, Somayeh Tourani, Hasan Sadeghi, Abbass Abolghasemi

**Affiliations:** 1 Department of Psychology, Islamic Azad University, Branch of Karaj, Karaj, IR Iran; 2 Department of Psychology, Allameh Tabatabaei University, Tehran, IR Iran; 3 Department of Psychology, Science and Research University, Young Researchers Club and elites, Ardabil, IR Iran; 4 Department of Psychology, University of Mohaghegh Ardabili, Ardabil, IR Iran

**Keywords:** Substance addiction, Anxiety, Depression, Stress, Psychological

## Abstract

**Background:**

Stressful life events may cause initiation of drug use among people. The main purpose of this study was to evaluate the effectiveness of stress management skill training on depression, anxiety and stress levels in drug addicts after withdrawal.

**Objectives:**

The population included all drug addicts after withdrawal in 2012 in Alborz province.

**Materials and Methods:**

The study was quasi-experimental with pretest-posttest design with a control group. Levels of emotional reactions (depression, anxiety and stress) in all referrals to a counseling center for drug withdrawal in 2012 using the Depression, Anxiety, Stress (DASS-21) questionnaire was assessed. The study population included drug addicts after withdrawal. The sampling method was available sampling and random assignment. Thirty people who had higher emotional reactions were randomly selected and divided into two test (n = 15) and control (n = 15) groups. For the test group, a stress management skill training course was held in twelve 90-minute sessions, but the control group received no intervention. The obtained data were analyzed using SPSS-19 software with analysis of covariance.

**Results:**

The results showed that stress management skill training has a significant effect on reducing emotional reactions (P < 0.01). It was noted that after 2 months test group follow-up, stress management training has retained its effect.

**Conclusion:**

Apparently, training addicts about life skills, particularly stress management seems to be a good idea.

## 1. Background

Addiction is a chronic disease whose emergence and persistence are influenced by genetic, psychological, social and physical factors. Therefore, the identification, prevention and treatment should encompass all these aspects. Addiction is a major problem in modern society; a problem that has destroyed millions lives. National macro investments have been made to deal with, treat it and to alleviate trauma. Every day, more people turn to drug and suffer its physical, psychological, cultural, familial, social and economic consequences. 

Several studies have been revealed on psychological causes of addiction and relapse after treatment. One factor that has been cited in many studies is stress (1). Stress symptoms such as depression, anxiety, nervous stress and tension, insomnia, sexual disorders, fatigue, reduce memory and lead to various interactional, physical and organic impairments such as chronic headaches; intestinal swelling, shortness of breath, etc. can be seen at the individual level. Today, stress is considered an inevitable part of human life. The mentioned studies on stress have emphasized what healthy behaviors is not occurring in a stress-free environment, the individual's assessment coping methods with stress and how to deal with it (2). 

Addiction has major affects not only on the addict, also, on the other family members. Possible negative effects of addiction on beloved members of addict friends and family are not easily measured and included emotional stress, financial costs, fatigue, anxiety and depression (3).

Emotional climate in families of people with substance abuse (e.g. conflict, instability or irritability) may increase vulnerability for drug-related problems in children and important people in life (4). Finally, the Opponent Process theory is another approach that seeks to explain the emergence and persistence of abuse behavior. According to this theory, drug is primarily used for gaining psychological and physiological pleasure. Overtime, as withdrawal effects become most severe and unpleasant, the patient reduces long-term drug withdrawal. At the end, according to this theory, the emergence of drug dependence often is used primarily to relieve negative symptoms of withdrawal (5).

Moreover, psychopathology is a risk factor for emergence and persistence of serious drug-related problems (6). There are many objective findings which indicate that psychological factors interfere with chronic diseases preparation, leading to exacerbated or improved disease process. Psychological factors also affect the disease symptoms and control. Psychological conditions such as stress, anxiety, depression, anger, frustration and negative emotions are identified as catalytic agents for health problems, and decreasing quality of life (7).

Cognitive-behavioral therapy (CBT) is one of the most frequent treatment studies for drug use disorders. So it is often used as a comparing or reference method in clinical experimental situations to evaluate other methods. The CBT goal is to clear the individual using on strategic skills and multi-faceted skills training in various fields. Cognitive behavioral strategies include decreasing referrals, decreasing drug symptoms, preparing for coping with stressful situations of relapse and nurturing the individual to stop using drug. Through discovering negative consequences of its continued use, self-regulation for identifying high-risk situations, identifying and addressing drug-related thoughts, and apparent recognition of relevant decisions that can lead the individual towards high-risk situations. Perhaps the most important treatment effect of CBT in addicts is to prevent relapse.

Most psychotherapy methods with methadone use cognitive-behavioral therapy whose purpose is to prevent relapse. In the same way, many cognitive behavioral techniques are used for decreasing patients' depression and increasing their motivation (3).

From cognitive-behavioral perspective, addiction is caused by a complex contact between cognitive, behavioral, emotional, familial-social, cultural processes and biological-psychological processes. So, the treatment consists of different aspects of entering into the inefficient system. However, CBT makes more emphasis on cognitive processes. Cognitive processes involve different activities such as thoughts, beliefs, ideas, schemas, values, opinions, expectations and assumptions. From this viewpoint, cognitive processes such as self-efficacy and locus of control are the therapeutic target. The cognitive behavioral pattern of drug use is used as compensatory strategies to regulate and deal with negative or unpleasant stressful. In Chau et al. research (8) and Kreek et al. (9) found that cognitive-behavioral integrated treatment (CBIT) training can reduce drug use by determining Through exercises three factors among drug abusers. 

In Jennifer research (10), coping and motivational style for cessation and reduction of alcohol used. They found that when a person is trained on coping strategies, he/she shows less reaction to powerful stimulus of drinking alcohol and the resultant stress. Nancy Pritzker and Ernest Gallo (2010) found that drug use and acute stress both considerably affect dopamine neuron synapses in midbrain and stimulate it. The results of this study also suggest that the stimulation of dopamine neurons synapses may contribute to drug use and the resultant stress. They also (9) found that genetic diversity affects personality and physical characteristics such as stress responses, risky and impulsive behaviors. It can be said that such individuals are candidates for drug-related diseases. Moreover, personality and physical characteristics have different effects on different stages of addiction which will cause its initiation, continuity, dependence and relapse. Addicts suffer from anxiety, depression, boredom, lack of morale, go towards addiction to get rid of the pains and to gain better feeling. From a cognitive-behavioral viewpoint, alcohol use and drug dependence are learned behaviors that are acquired through experience, and hence learning processes play an important role in the emergence and persistence of addiction and drug dependence. If drug use leads to desired results such as good feeling and lower level of stress, its continuity can become an ordinary method for obtaining the same results (3).

Since drug consumption reduces stress in addicts, it seems that stress management skill training technique can reduce pain, mental depression, anxiety and stress of addicts. So this question is posed: whether stress management skill training is effective on reducing emotional reactions? In this regard, this hypothesis was proposed: stress management skill training is effective on reducing emotional reactions among drug addicts after drug withdrawal. 

## 2. Objectives

The population included all drug addicts after withdrawal in 2012 in Alborz province.

## 3. Materials and Methods

The method was quasi-experimental type with pretest-posttest design with a control group. The population included all drug addicts after withdrawal in 2012. This study used available sampling and random assignment. Thus, everyone who referred to an addiction counseling center in Alborz province was assessed for emotional reactions using DASS-21 questionnaire. Then 30 patients who had higher emotional reactions than average were randomly selected and divided into two test (n = 15) and control (n = 15) groups. The subjects were initially pretested. A group training course was hold on stress management skills for the test group, in twelve 90-minute sessions during six weeks, whiles the control group received no intervention. The provisions of these sessions were set using stress management approach and were approved by psychologists. Training sessions are summarized as follows: First session, introducing members to each other expressing the group rules and programs that are going to be executed for this period; Second session, stress coping strategies (problem-focused and emotion-based methods); Third session, fractional muscle relaxation method training (relaxation); Fourth session, courage behavior (introduction and training of courage behavior, say no and role playing). Fifth session, problem solving (problem solving definition and expression, the use of examples and instances); Sixth session, coping with irrational thoughts (identification and detection of cognitive thoughts and errors); Seventh session, coping skills (changes in attitude and internal perceptions, how to interact with the environment and to increase physical abilities); Eighth session, time management (goal setting, planning, time saving and avoid wasting time); Ninth session, positive thinking (changing negative attitude towards positive thoughts and expressing positive emotions); Tenth session, nutrition and exercise (lifestyle change and give importance to nutrition); Eleventh session, self-esteem enhancement (identifying the individual's characteristics, hearing his/her characteristics from another person); Twelfth session, conducting posttest on the test and control groups. Finally, to assess the effect of stress management skill training program in a longer term, follow up study was conducted after two months for the test group. SPSS19 software was used and analysis of covariance was done for data analysis.

### 3.1. Tools

DASS-21 Questionnaire: the DASS-21 scale was used for data collection. The questionnaire included 21 questions. Seven questions were considered for each emotional state (anxiety, depression and stress). The questionnaire was designed in two 41- and 42-question versions (11). The 21-question form is its shortened model. The scale has adequate adaptive and differential validity and has been used in different studies. In a large sample size (n = 717 students), (12) showed that the Beck depression inventory (r = 0.74) is highly correlated with the DASS scale. In addition, (13) obtained a similar clinical pattern for correlation effect in clinical specimens.

In a sample of 1771 British, Crawford and Henry also compared tool with two other for depression and anxiety, and reported its reliability with Cronbach's alpha for depression 0.95, anxiety 0.90, stress 0.93 and for total score 0.97. In Iran, Samani and Jokar reported the retest reliability for depression, anxiety and stress scales 0.80, 0.76 and 0.77, respectively. They also reported Cronbach's alpha for depression, anxiety and stress, 0.81, 0.74 and 0.78, respectively. To examine the validity of this scale was used factor analysis methods. The KMO index value was 0.902. The index value was 3093.93 in the Bartlett's Sphericity test which was significant at the level of 0.0001. In the present study, Cronbach's alpha was 0.71 which shows that it has a good internal consistency. Each sub-scale (in DASS-21) contains seven questions and scores of each question is calculated through summing the scores of the questions. Each question has a 4-point Likert scale from never to completely.

## 4. Results

Table 1 shows descriptive information for variables such as average and standard deviation. It shows descriptive properties of the study variables in the pretest, posttest and follow-up test based on the studied groups (control and test). 

**Table 1.** Statistical Properties for Variables in the Pretest, Posttest and Follow-Up Test Based on the Studied Groups

	Number	Pre-test, Mean ± SD	Post-test, Mean ± SD	Follow-up, Mean ± SD
**Depression**				
Control	15	17.11 ± 3.4	17.16 ± 3.2	17.02 ± 3.2
Test	15	17.31 ± 3.4	14.28 ± 3.5	13.9 ± 2.94
**Anxiety**				
Control	15	13.16 ± 2.6	13.12 ± 3	12.8 ± 2.43
Test	15	13.22 ± 2.7	11.31 ± 2.3	10.42 ± 2.81
**Stress**				
Control	15	21.16 ± 3.5	22.41 ± 3.45	22.05 ± 3.80
Test	15	21.14 ± 3.5	18.25 ± 3.1	17.2 ± 3.2

Analysis of covariance was used to evaluate the effect of stress management skill training on reducing depression, anxiety and stress levels in drug addicts. Using analysis of covariance allows the examination of effects (pretest-posttest). In analysis of covariance, the main effect of the group is used to evaluate the precise effect of experimental intervention on the subjects of the two groups. In other words, with observing the main effect of the group, the researcher concluded that any observed difference in dependent variable in the two test group is significant compared with the control group. It is also considered as the best method. In this method, posttest averages are compared after adjusting pretest scores (14). About the reason for using the analysis of covariance test instead of differential scores, it can be noted that if the effect of the independent variable is not so strong, due to unreliability of score differences, using the t test method is not recommended, because scores must be reliable enough to reflect differences and determine the difference between the test group and the control group. In these schemas, for data analysis using covariance method, it is better to remove the effect of pretest scores from posttest scores and then the average of these residualized or regressed scores are compared on the control and test groups (15).

In analysis of covariance usually seven common hypotheses are considered (16). Before statistically calculating the "independence of scores" hypothesis, the following were observed: normality test of variables using Kolmogroff-Smirnov (KS) test, linearity using test for linearity at (P < 0.01), independency of the associated variable and the experimental procedure as well as measuring the associated variable without error in completely controlled conditions. Homogeneity of variances was examined using SPSS software and results of homogeneity of regression slopes are discussed next. According to the Levine test, the hypothesis of equal variances was confirmed for depression (F = 1.23 and P = 0.28), anxiety (F = 3.38 and P = 0.07) and stress (F = 3.87 and P = 0.06). Homogeneity of regression slopes, was calculated using the regression homogeneity test, the F ratio was 2.58 which was smaller than the critical F (4.22) and was non-significant at (P < 0.05), so the hypothesis of homogeneity of regression slopes was also confirmed and there were assumptions required for statistical test. 

The main effects of the variables are reported in Table 2. Based on these results, the obtained F value for depression (20.63), anxiety (90.71) and stress (94.30) variables are significant at (P < 0.01), it can be concluded that stress management skill training has had a significant impact on reducing emotional reactions. Moreover, since the observed power and the effect size are high in the variable, one can certainly conclude that the experimental intervention are effective on the variables. 

**Table 2.** Summary Results of Analysis of Covariance for the Studied Variables in the Post-test Stage

	SS^*^	df	MS^*^	F	P	Observed Power	Eta-squared
**Depression**	7458.45	1	7458.45	20.63	0.0001	0.99	0.74
**Anxiety**	43236.81	1	43236.81	90.71	0.0001	1.00	0.77
**Stress**	44356.06	1	44356.06	94.30	0.0001	1.00	0.78

^*^Abbreviations: SS, sum of squares; MS, Mean Square

Table 3 shows comparison results of analysis of covariance of pretest and follow-up test (second post-test) to evaluate the durability of the experiment effect. The main effects of variables are reported in Table 3. According to these results, since the F values for depression (18.63), anxiety (76.71) and stress (54.30) are significant at P < 0.01, and comparison of averages of depression, anxiety and stress in the pretest and the follow-up test (second posttest), can conclude that the scores of depression, anxiety and stress in the follow-up test have a considerable stability (Table 1). So, stress management skill training cause stability in reduced emotional reaction in addicts after drug withdrawal. 

**Table 3.** Summary Results of Analysis of Covariance for the Studied Variables in the Follow-Up Stage

	SS^*^	df	MS^*^	F	P Value	Observed Power	Eta-squared
**Depression**	6342.45	1	6342.45	18.63	0.0001	1.00	0.87
**Anxiety**	34231.81	1	34231.81	76.71	0.0001	1.00	0.86
**Stress**	45654.09	1	45654.09	54.30	0.0001	1.00	0.65

^*^Abbreviations: SS, sum of squares; MS, Mean Square

## 5. Discussion

This study was aimed to examine the effects of stress management skill training on depression, anxiety and stress levels in addicts after drug withdrawal in the addiction withdrawal clinics. As noted in the results section, the findings indicated that stress management skill training programs have an impact on anxiety, depression and stress levels in those who withdrew from drugs and decrease the emotions. These programs also cause stability in reduced emotional responses in addicts after drug withdrawal. The results of this study are consistent with previous studies (1, 3, 7, 17). On explaining the effectiveness of life skills training program on the subjects' negative emotions in this study, it can be said that life skills, on one hand, help people to know and identify their strengths and weaknesses and strive to control their weaknesses and strengths. The awareness of strengths and weaknesses helps the individual to be more efficient to find more appropriate ways to handle problems; as a result, reduce his/her stress. On the other hand, group training has a positive effect on reducing stress. Gathering of people in a group made individuals fully perceive that others have similar problems and could use the experiences of each other to deal with stress. This is very effective in reducing stress and increasing self-confidence. Fractional muscle relaxation training in the third session reduced subjects tension that they feel in distress and stressful situations makes people to reduce muscle tension. By training courage in behavior to say No, teach individuals to resist against drug use and cope with the resultant stress using the methods learned during the course, can be hopeful about effectiveness of training in addicts after drug withdrawal. During stress management training programs, the individual tries to dominate the stressful factor with removing or reducing adverse effects of stress and using problem-focused coping method in stressful situations. Examination of stress management training content have an important role in reducing emotional reactions; given that extremely stressful events highly affects drug and alcohol use, and stress can cause a relapse in people who have withdrawn from drug. Numerous studies have proven that there is a close relationship between stress and addiction, especially in times of back slip, stress plays an important role in increasing the individual's vulnerability to drug reuse. Stress may even make the individual turn to drug reuse after a long period of withdrawal (18). Considering that there is a close relationship between depression and addiction in adults (19). Depressed patients try to change their feeling by self-medication which is a tendency to addiction. It seems that stress management training can be effective on the prevention of back slip and relapse in addicts who have withdrawn from drug because those who have anxiety-related symptoms turn to self-medication with drug and alcohol. This increases the risk of problems arises from use of substance (18). Overall, based on the results of this study and those obtained by other researchers, stress management skill training can decrease depression, anxiety and stress and since these negative emotional reactions affect other aspects of life and also reduce relapse in drug users who have withdrawn from drug, it seems that stress control and management can be effective on improving mental health and quality of life over time. Since this study was conducted in an addiction withdrawal center in Karaj, one should be cautious in generalizing its results. It is recommended that the same study to be conducted in other regions of the country. To make consistency between the results, these could be generalized including holding life skills training, especially stress management, along with other issues and educational programs by authorities on preschool level to provide a primary prevention program.
